# An anatomical study for the location of suprascapular and spinoglenoid notches using three-dimensional computed tomography images of scapula

**DOI:** 10.1016/j.jseint.2022.04.002

**Published:** 2022-05-05

**Authors:** Kazumasa Takayama, Hiromu Ito

**Affiliations:** Department of Orthopaedics, Kurashiki Central Hospital, Kurashiki, Okayama, Japan

**Keywords:** Suprascapular nerve, Spinoglenoid notch, Suprascapular notch, Posterolateral corner of the acromion, Secondary ossification center, Ganglion

## Abstract

**Background:**

The optimal position for creating portals for arthroscopic suprascapular nerve decompression has not been sufficiently verified. Therefore, this study aimed to investigate the anatomical characteristics of the scapula for optimal portal creation using 3-dimensional computed tomography images. The posterolateral corner of the acromion was designated as the starting point for measurements because there is no secondary ossification center present.

**Methods:**

This study included 223 patients (females, 129; males, 94) who underwent computed tomography of the shoulder joint. Three-dimensional images of the scapula were created, and the distance from the posterolateral corner of the acromion to the suprascapular and spinoglenoid notches was measured. Additionally, the correlation coefficient with height and the differences between the female and male groups were investigated.

**Results:**

The distances from the posterolateral corner of the acromion to the suprascapular and spinoglenoid notches were 42.9 ± 4.6 and 31.5 ± 3.6 mm, respectively, and their correlation coefficients with height were 0.12 and 0.067, respectively. There was no significant difference in the distance from the posterolateral corner of the acromion to the suprascapular (42.5 ± 4.1 vs. 43.9 ± 5.1 mm, *P* = .098) and to the spinoglenoid (31.4 ± 3.3 mm vs. 32.0 ± 3.9 mm, *P* = .12) notches between the female and male groups.

**Conclusion:**

Regardless of height and sex, the distances from the posterolateral corner of the acromion to the suprascapular and spinoglenoid notches were approximately 43 and 32 mm, respectively. Therefore, creating portals at these locations may be effective for arthroscopic suprascapular nerve decompression.

The suprascapular nerve originates from the ventral rami of cervical roots C5 and C6 in 76% of the general population; C4, C5, and C6 nerve roots in 18%; and C5 nerve roots in 6%.^16^ It controls the movement of the supraspinatus and infraspinatus muscles^2^^,^^11^^,^^17^^,^^18^ and has an intrinsic sensory area on the dorsal side of the scapula.^3^^,^^13^ Occasionally, the suprascapular nerve gets entrapped at the suprascapular and spinoglenoid notches.^8^ Arthroscopic dissection of the superior transverse scapular ligament for entrapment neuropathy around the suprascapular notch has been reported,^9^ and several anatomical studies have reportedly described the optimal location for creating portals.^1^^,^^9^^,^^19^ In contrast, entrapment of the suprascapular nerve at the spinoglenoid notch is often secondary to a labrum injury.^20^ Ganglion cysts often occur in association with labrum injury and can be removed by manipulation with an intra-articular approach.^2^^,^^4^^,^^7^^,^^8^^,^^22^ However, the ganglion cyst is sometimes not contiguous to labral injury and instead occurs solitarily around the spinoglenoid notch,^10^ making decompression using an intra-articular approach difficult. An arthroscopically assisted approach through the subacromial space for the decompression of the suprascapular nerve at the spinoglenoid notch has been reported.^6^^,^^21^ However, there have been only a few anatomical studies on the location of the spinoglenoid notch^14^^,^^15^; therefore, the optimal position for creating a viewing or working portal has not been sufficiently verified. Anatomical landmarks that are usually palpable on the body surface of the scapula during the arthroscopic surgery include the medial border of the acromion, posterolateral corner of the acromion, lateral border of the acromion, and anterior corner of the acromion. Therefore, we believe that it would be clinically useful to determine the distance between these palpable landmarks and the suprascapular and spinoglenoid notches when performing an arthroscopic nerve decompression surgery.

This study aimed to investigate the morphology of the scapula by measuring 1:1-scale 3-dimensional images of the scapula and to identify the anatomical characteristics required for optimal portal creation. In addition, the validity of portal location in previous studies was verified.^9^^,^^14^^,^^15^^,^^19^ The bone size is considered to be related to height; however, when we measured the distance from the posterolateral corner of the acromion to the spinoglenoid notch preoperatively in patients with spinoglenoid ganglion cysts, the results showed that the distance was nearly constant regardless of height. Therefore, the proposed hypothesis of this study was that the distance from anatomical landmarks such as the posterolateral corner of the acromion to the suprascapular and spinoglenoid notches would be almost constant regardless of the patient’s height.

## Materials and methods

### Study design and patient selection

This retrospective cohort study included patients who underwent computed tomography (CT) of the shoulder joint at a single institution between April 2018 and October 2021.

This study was approved by the institutional review board. Written informed consent was obtained from all patients prior to enrollment. A total of 242 patients underwent CT of the shoulder joint during the study period. Patients in whom the entire scapula was not imaged (2 cases) and those with fractures in the scapula (2 cases) or arthritic changes in the glenohumeral joint (15 cases) were excluded.

CT images were obtained using Canon Aquilion Prime SP 80-row multidetector CT scanner (Canon Medical Systems Corporation, Tokyo, Japan) following the standard shoulder protocol of our institution (120 kV, Adaptive Iterative Dose Reduction using three-dimensional processing, Canon Medical Systems Corporation) with a reconstruction thickness of 0.5 mm. An image-conversion software program was used (Ziostation2; Ziosoft Inc., Tokyo, Japan) to create 3-dimensional images of the scapula from 2-dimensional image data (Digital Imaging and Communications in Medicine), which were used for measurements.

### Measurement items and outcome assessment

**Figure 1:**
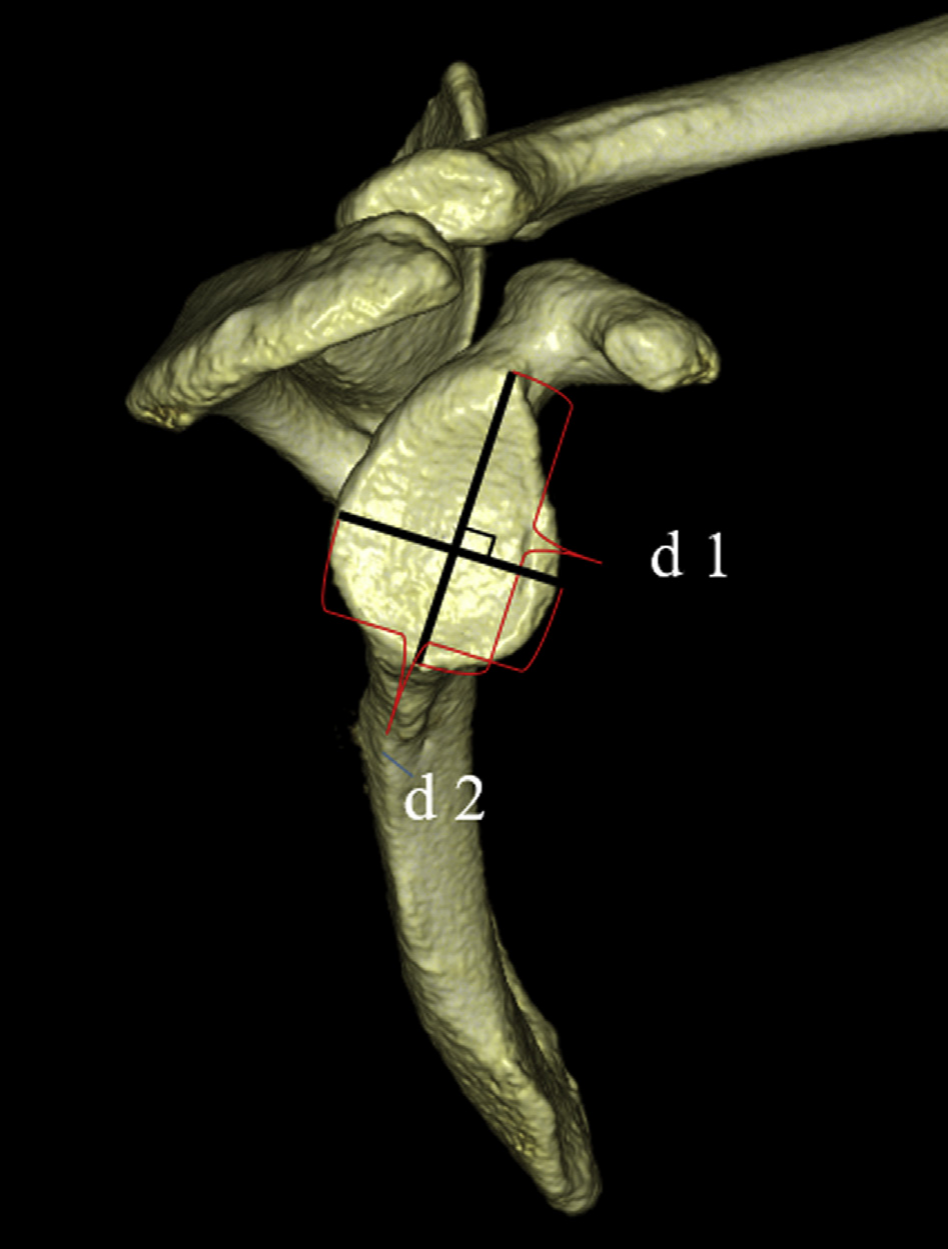
(*d 1*) The longitudinal diameter is defined as the distance from the supraglenoid tubercle to the infraglenoid tubercle. (*d 2*) The transverse diameter is defined as the maximum distance on the glenoid, perpendicular to the line connecting the supraglenoid and infraglenoid tubercles.

Baseline characteristics—age, sex, height, weight, and body mass index (BMI)—were obtained from the medical records. In addition, the longitudinal and transverse diameters of the glenoid were investigated. The longitudinal diameter was defined as the distance from the supraglenoid tubercle to the infraglenoid tubercle (d 1). The transverse diameter was defined as the maximum distance on the glenoid, perpendicular to the line connecting the supraglenoid and infraglenoid tubercles (d 2) (Fig. 1).

The distance from the posterolateral corner of the acromion to the midpoint of the medial border of the scapular spine was defined as the maximum transverse diameter of the scapula (d 3).

**Figure 2:**
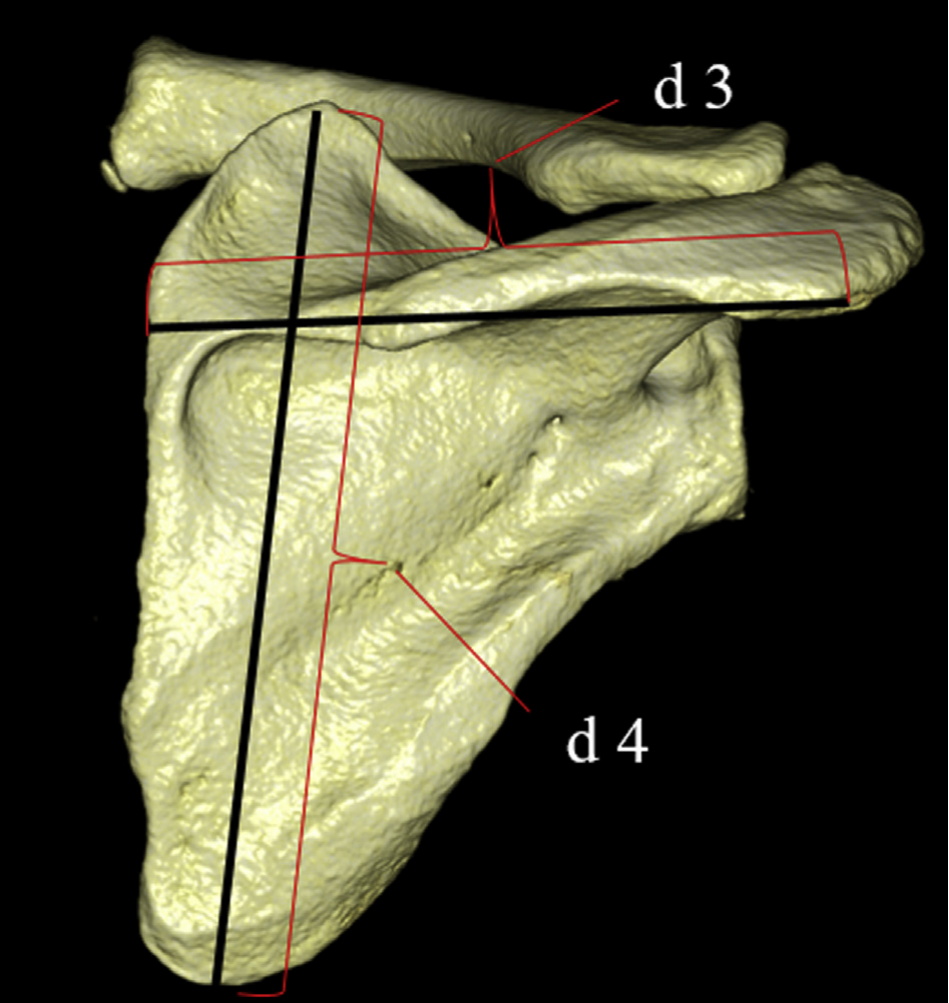
(*d 3*) The distance from the posterolateral corner of the acromion to the midpoint of the medial border of the scapular spine is designated as the maximum transverse diameter of the scapula. (*d 4*) The distance from the superior angle to the inferior angle is measured as the maximum longitudinal diameter of the scapula.

The distance from the superior angle to the inferior angle was defined as the maximum longitudinal diameter of the scapula (d 4) (Fig. 2).

**Figure 3:**
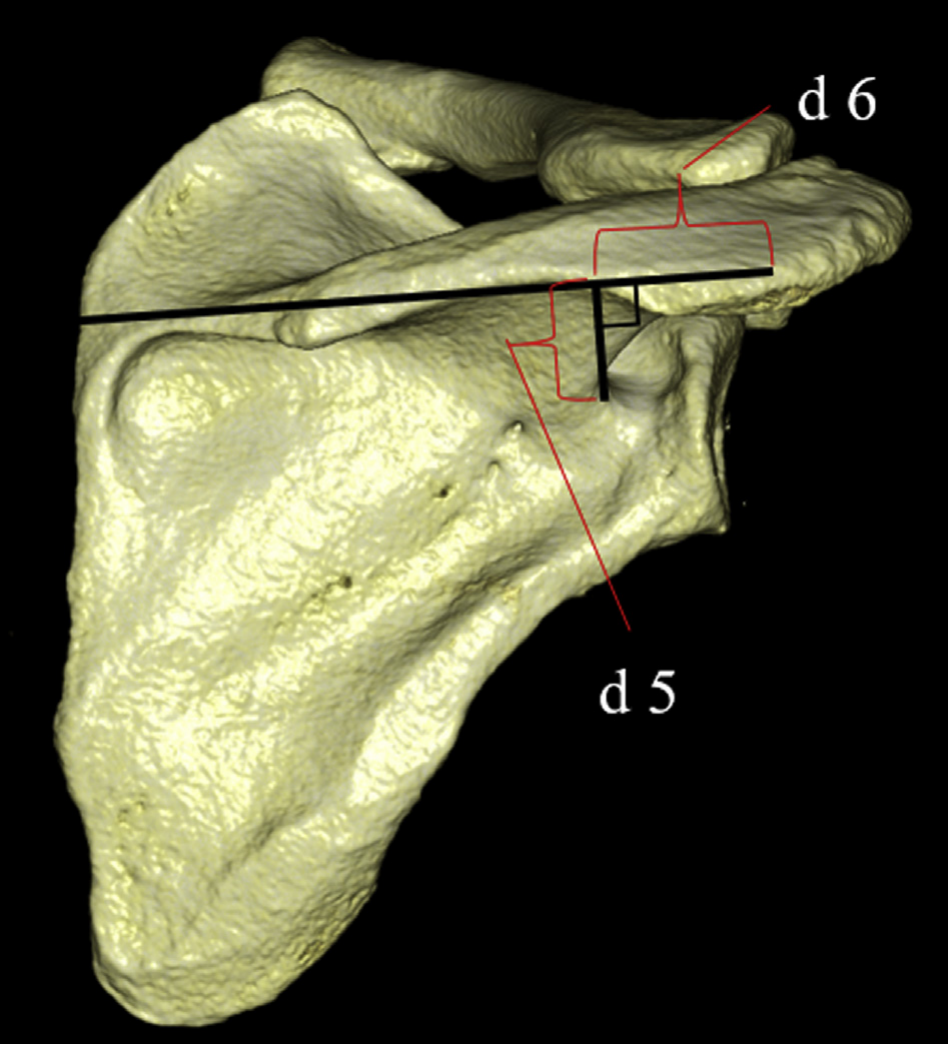
(*d 5*) A perpendicular line is drawn from the spinoglenoid notch to the line connecting the posterolateral corner of the acromion and the midpoint of the medial border of the scapular spine, and the distance between the intersection point and spinoglenoid notch is measured. (*d 6*) A perpendicular line is drawn from the spinoglenoid notch to the line connecting the posterolateral corner of the acromion and the midpoint of the medial border of the scapular spine, and the distance between the intersection point and posterolateral corner of the acromion is measured.

**Figure 4:**
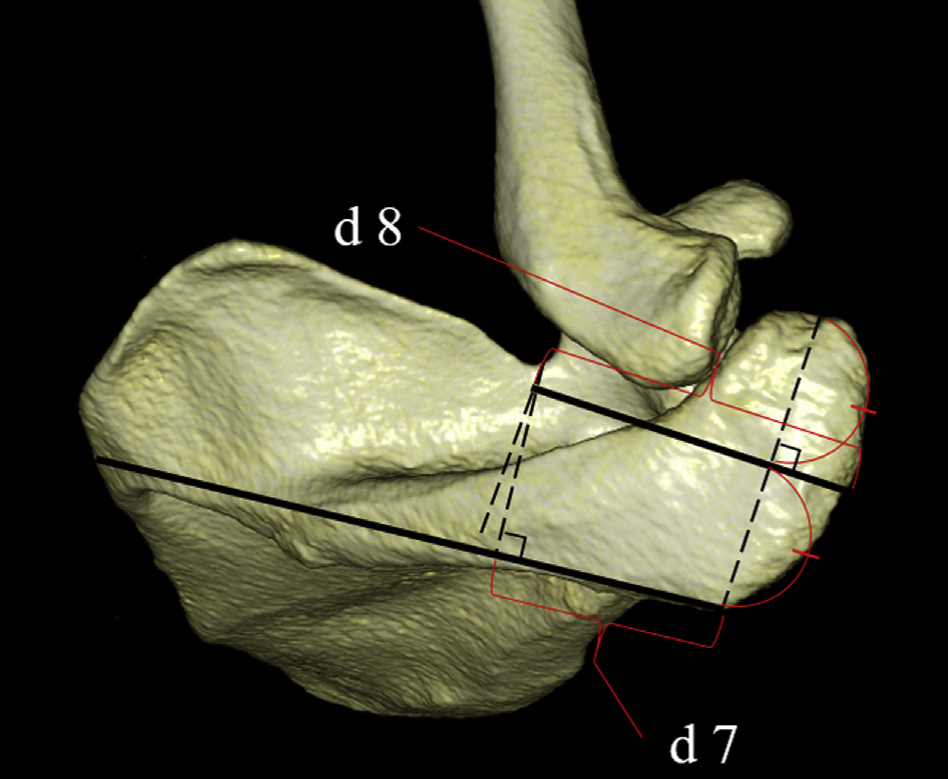
(*d 7*) A perpendicular line is drawn from the suprascapular notch to the line connecting the posterolateral corner of the acromion and the midpoint of the medial border of the scapular spine, and the distance between the intersection point and posterolateral corner of the acromion is measured. (*d 8*) A perpendicular line is drawn from the suprascapular notch to the perpendicular bisector of the lateral border of the acromion, and the distance between the intersection point and lateral border of the acromion is measured.

A perpendicular line was drawn from the spinoglenoid notch to the line connecting the posterolateral corner of the acromion and the midpoint of the medial border of the scapular spine, and the distances between the intersection point and spinoglenoid notch (d 5) and between the intersection point and posterolateral corner of the acromion (d 6) were measured (Fig. 3). A perpendicular line was drawn from the suprascapular notch to the line connecting the posterolateral corner of the acromion and the midpoint of the medial border of the scapular spine, and the distance between the intersection point and posterolateral corner of the acromion was measured (d 7) (Fig. 4). A perpendicular line was drawn from the suprascapular notch to the perpendicular bisector of the lateral border of the acromion, and the distance between the intersection point and lateral border of the acromion was measured (d 8) (Fig. 4).

The normality of these measurements was evaluated, and 95% confidence intervals were investigated. The correlation coefficients between distance and height were also investigated. Moreover, these patients were divided into female and male groups, and the following variables were compared between the groups: age, height, weight, BMI, and d 1–8.

### Statistical analyses

The means of these continuous variables—length, height, weight, BMI, and age—were compared using the Mann-Whitney U test, and *P* < .05 was considered significant. The Shapiro-Wilk normality test was used to evaluate normality, and a Spearman’s rank correlation coefficient was used to evaluate the correlation coefficient (0.7–1.0, strongly correlated; 0.4–0.7, correlated; 0.2–0.4, weakly correlated; and 0–0.2, no correlation).

All statistical analyses were conducted using EZR (Saitama Medical Center, Jichi Medical University, Saitama, Japan), a modified version of the R commander (The R Foundation for Statistical Computing, Vienna, Austria).

## Results

A total of 223 patients (females, 129; males, 94) were included in this study. Their baseline characteristics are shown in Table Ⅰ. The mean age of the participants was 68.2 ± 18.0 (range, 16–93) years, which was relatively old. More than half of the patients had humeral surgical neck fractures. The result for each distance (d 1–8) and their correlation coefficients with height are shown in Table II. The d 2, 3, 6, 7, and 8 showed normal distribution. The maximum transverse diameter of the scapula (d 3) was correlated with the height (correlation coefficient, 0.60). The longitudinal diameter of the scapula (d 4) was strongly correlated with height (correlation coefficient, 0.72). Conversely, d 5–7 were not correlated with height (correlation coefficient d 5, 0.16; d 6, 0.067; and d 7, 0.12), whereas d 8 was correlated with height (correlation coefficient, 0.55).

**Table I:** Baseline characteristics of the patients.

Sex	Female: 129 cases; male: 94 cases
Age, yr	68.2 ± 18.0, range: 16–93 (65.8–70.6)
Height, cm	155.9 ± 10.2, range: 131.7–183.9 (154.5–157.3)
Body weight, kg	57.1 ± 12.9, range: 25.1–116.0 (49.4–64.8)
Body mass index	23.4 ± 4.0, range: 12.9–42.1 (22.9–23.9)
Diagnosis, cases	Humeral surgical neck fracture: 118 Humeral shaft fracture: 11 Clavicle fracture: 31 Tuberosity fracture: 20 Rotator cuff tear: 14 Shoulder dislocation: 20 Others: 9

Continuous variables are presented as mean ± standard deviation. Ninety-five-percent confidence intervals are presented in parentheses.

**Table II:** Value for each distance and its correlation coefficient with height.

Height, cm		155.9 ± 10.2, range 131.7–183.9 (154.51–57.3)∗
Distance		Correlation coefficient with height
d 1	41.8 ± 3.6, range 34.2–54.1 (41.3–42.3)	0.53
d 2	30.6 ± 3.7, range 22.1–41.4 (30.1–31.1)∗	0.40
d 3	107.6 ± 7.5, range 85.1–129 (106.6–108.6)∗	0.60
d 4	148.2 ± 12.8, range 123.81–99.6 (146.5–149.9)	0.72
d 5	17.8 ± 2.4, range 12.2–25.2 (17.5–18.1)	0.16
d 6	31.5 ± 3.6, range 21.2–40.5 (31.0–32.0)∗	0.067
d 7	42.9 ± 4.6, range 26.4–53.6 (42.3–43.5)∗	0.120
d 8	61.3 ± 4.0, range 45.4–69.4 (60.8–61.8)∗	0.550

Continuous variables are presented as mean ± standard deviation. Ninety-five-percent confidence interval are presented in parentheses.

d 1: Distance from the supraglenoid tubercle to the infraglenoid tubercle.

d 2: Maximum distance on the glenoid, perpendicular to the line connecting the supraglenoid and infraglenoid tubercles.

d 3: Distance from the posterolateral corner of the acromion to the midpoint of the medial border of scapular spine.

d 4: Distance from the superior angle to the inferior angle.

d 5: A perpendicular line was drawn from the spinoglenoid notch to the line connecting the posterolateral corner of the acromion and the midpoint of medial border of the scapular spine, and the distance between the intersection point and the spinoglenoid notch.

d 6: A perpendicular line was drawn from the spinoglenoid notch to the line connecting the posterolateral corner of the acromion and the medial border of the scapula, and the distance between the intersection point and the posterolateral corner of the acromion.

d 7: A perpendicular line was drawn from the suprascapular notch to the line connecting the posterolateral corner of the acromion and the midpoint of medial border of the scapular spine, and the distance between the intersection point and the posterolateral corner of the acromion.

d 8: A perpendicular line was drawn from the suprascapular notch to the perpendicular bisector the lateral border of acromion, and the distance between the intersection point and the lateral border of acromion.

∗ Normal distribution.

### Comparison of the measured variables between female and male participants

The results of the comparison of the measured variables between female and male patients are shown in Table III. There was a difference of approximately 15 cm in height between the female and male groups. There were significant differences between the female and male groups in d 1, 2, 3, 4, and 8, where the correlation coefficient with height was >0.5. However, there were no correlation with height in d 6 and 7 and no significant difference between the female and male groups. Additionally, the 95% confidence intervals in d 6 (30.8–32.0 vs. 31.2–32.8) and 7 (41.8–43.2 vs. 42.8–45.0) were similar in the female and male groups. Moreover, d 5 was not correlated with height, and the mean difference between the 2 groups was approximately 1 mm (17.4 ± 2.0 vs. 18.4 ± 2.8, *P* = .017). Although there was a statistically significant difference in d 5, the difference was 1 mm, which clinically may be likely irrelevant in terms of portal placement.

**Table III:** Comparison of the baseline and measured distances in the female and male patients.

	Female (n = 129)	Male (n = 94)	*P* value
Age, yr	73.1 ± 14.6	61.2 ± 19.7	<.001∗
Height, cm	149.9 ± 6.4, range 131.7–168.0 (148.8–151.0)	165.1 ± 7.3, range 146.0–183.9 (163.6–166.6)	<.001∗
Weight, kg	52.8 ± 10.4, range 25.0–100.0 (51.0–54.6)	63.9 ± 13.5, range 41.0–116.0 (61.0–66.8)	<.001∗
Body mass index	23.5 ± 4.1, range 12.9–40.0 (22.8–24.2)	23.3 ± 4.1, range 16.9–42.1 (22.4–24.2)	.300
d 1	39.6 ± 2.9, range 34.2–48.3 (39.1–40.1)	44.8 ± 3.2, range 37.8–54.1 (44.1–45.5)	<.001∗
d 2	29.0 ± 3.1, range 22.0–38.1 (28.5–29.5)	33.1 ± 3.3, range 26.4–41.4 (32.4–33.8)	<.001∗
d 3	104.1 ± 5.9, range 85.1–119.6 (103.1–105.1)	113.0 ± 6.6, range 90.0–129.0 (111.6–114.4)	<.001∗
d 4	141.3 ± 7.9, range 123.8–166.4 (139.9–142.7)	159.4 ± 11.7, range 139.5–199.6 (156.8–162.0)	<.001∗
d 5	17.4 ± 2.0, range 12.2–22.2 (17.0–17.8)	18.4 ± 2.8, range 13.1–25.2 (17.8–19.0)	.017†
d 6	31.4 ± 3.3, range 22.6–40.5 (30.8–32.0)	32.0 ± 3.9, range 21.2–40.4 (31.2–32.8)	.120
d 7	42.5 ± 4.1, range 27.7–51.0 (41.8–43.2)	43.9 ± 5.1, range 26.4–53.6 (42.8–45.0)	.098
d 8	55.7 ± 3.0, range 45.4–64.8 (55.2–56.2)	60.5 ± 3.3, range 51.5–69.4 (59.8–61.2)	<.001∗

Continuous variables are presented as mean ± standard deviation. Ninety-five-percent confidence intervals are presented in parentheses.

d 1: Distance from the supraglenoid tubercle to the infraglenoid tubercle.

d 2: Maximum distance on the glenoid, perpendicular to the line connecting the supraglenoid and infraglenoid tubercles.

d 3: Distance from the posterolateral corner of the acromion to the midpoint of the medial border of scapular spine.

d 4: Distance from the superior angle to the inferior angle.

d 5: A perpendicular line was drawn from the spinoglenoid notch to the line connecting the posterolateral corner of the acromion and the midpoint of medial border of the scapular spine, and the distance between the intersection point and the spinoglenoid notch.

d 6: A perpendicular line was drawn from the spinoglenoid notch to the line connecting the posterolateral corner of the acromion and the medial border of the scapula, and the distance between the intersection point and the posterolateral corner of the acromion.

d 7: A perpendicular line was drawn from the suprascapular notch to the line connecting the posterolateral corner of the acromion and the midpoint of the medial border of the scapular spine, and the distance between the intersection point and the posterolateral corner of the acromion.

d 8: A perpendicular line was drawn from the suprascapular notch to the perpendicular bisector the lateral border of acromion, and the distance between the intersection point and the lateral border of acromion.

∗ *P* < .01.

† *P* < .05.

## Discussion

### Summary of the results

The longitudinal and transverse diameters of the scapula were correlated with height. As we hypothesized, d 6 (the distance between the posterolateral corner of the acromion and spinoglenoid notch) and 7 (the distance between the posterolateral corner of the acromion and suprascapular notch) were almost constant regardless of the patient’s height and sex. Furthermore, d 5 (the distance between the scapular spine and spinoglenoid notch) was significantly different between the female and male groups; however, it was not correlated with height. The distance between the lateral border of the acromion and suprascapular notch (d 8) was significantly different between the female and male groups and correlated with height.

### Association between the secondary ossification center and each value

The scapula has at least 7 secondary ossification centers, comprising 1 subcoracoid center, 3 inferior glenoid centers, 1 acromion center, 1 inferior angle center, and 1 or more medial border centers.^5^^,^^12^^,^^23^ These secondary ossification centers were present in d 1–4 and 8 as measured in this study. They were all correlated with height and demonstrated significant differences between the female and male groups. In contrast, d 5–7 did not contain a secondary ossification center and were not correlated with height. There was no significant difference in d 6 and 7 between the female and male groups. If the distance from the starting point (where there is no secondary ossification center) to the spinoglenoid or suprascapular notch was measured, it may be possible to obtain values that are not affected by height.

The scapula is located on the posterolateral aspect of the thoracic cage and forms the back of the shoulder girdle; the superior angle is located at the approximate level of the first or second thoracic vertebra. In contrast, the inferior angle (the lowest part of the scapula) is located at the level of the seventh or eighth thoracic vertebra. Therefore, it is reasonable that the longitudinal diameter of the scapula (d 4) would have a strong correlation with height. Furthermore, d 3 was correlated with height because a secondary ossification center was present at the medial border of the scapula. Therefore, it may be unreasonable to use the medial border of the scapula as the starting point for measurement. In this study, the posterolateral corner of the acromion was used as the starting point for investigating the distance to the suprascapular and spinoglenoid notches because it does not contain a secondary ossification center. The distance from the scapular spine to the spinoglenoid notch (d 5), that from the posterolateral corner of the acromion to the spinoglenoid notch (d 6), and that from the posterolateral corner of the acromion to the suprascapular notch (d 7) were approximately 18, 32, and 43 mm, respectively. However, none of them were correlated with height; therefore, they can be considered indicators for creating a viewing or working portal.

### Assessing the optimal portal placement for suprascapular nerve decompression

**Figure 5:**
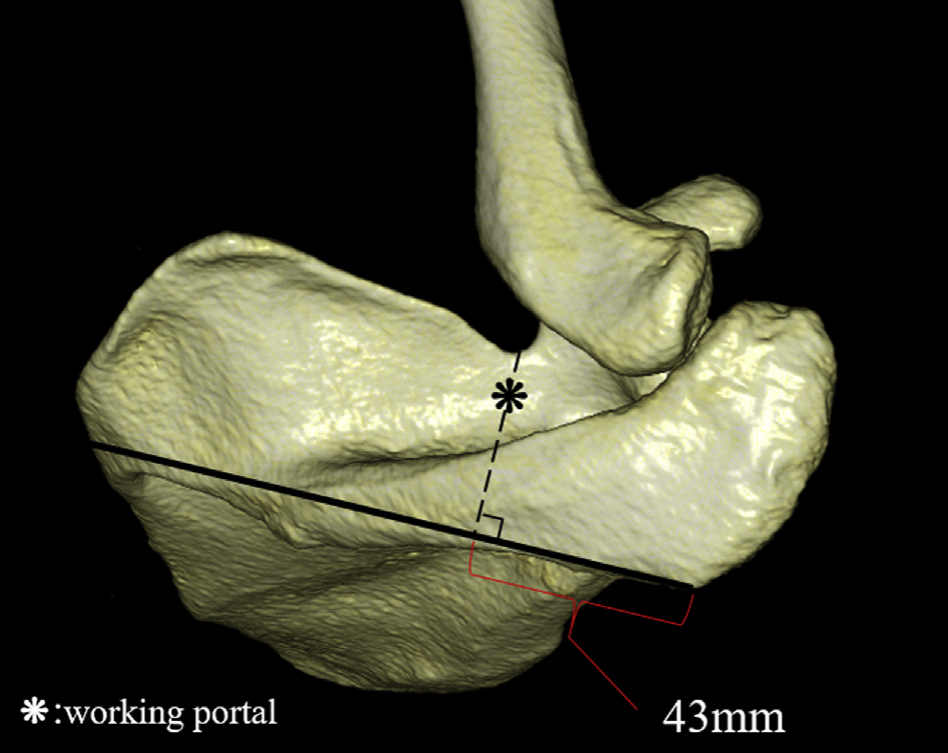
Following the approach used by Lafosse et al,^9^ it would be optimal to view from the lateral portal and create a working portal positioned between the clavicle and scapular spine, 43 mm medial to the posterolateral corner of the acromion.

A previous study reported that a portal was created approximately 7 cm medial to the lateral border of the acromion and used as a working portal for transecting the transverse scapular ligament,^9^ which corresponds to d 8 in our study; d 8 was correlated with height (correlation coefficient, 0.55) and demonstrated significant differences between the female and male groups. Owing to the presence of the secondary ossification center in the acromion,^23^ we believe that it is not necessarily correct to create a portal at 7 cm medial to the lateral border of the acromion. Warner et al^19^ reported that the suprascapular notch is 4.5 ± 0.5 cm from the posterolateral corner of the acromion, and our study (d 7) demonstrated similar results. Therefore, it was considered that approximately 43 mm medial to the posterolateral corner of the acromion could be a useful indicator for creating a portal to transect the transverse scapular ligament. Following the approach used by Lafosse et al,^9^ it would be optimal to view from the lateral portal and create a working portal positioned between the clavicle and scapular spine, 43 mm medial to the posterolateral corner of the acromion (Fig. 5).

**Figure 6:**
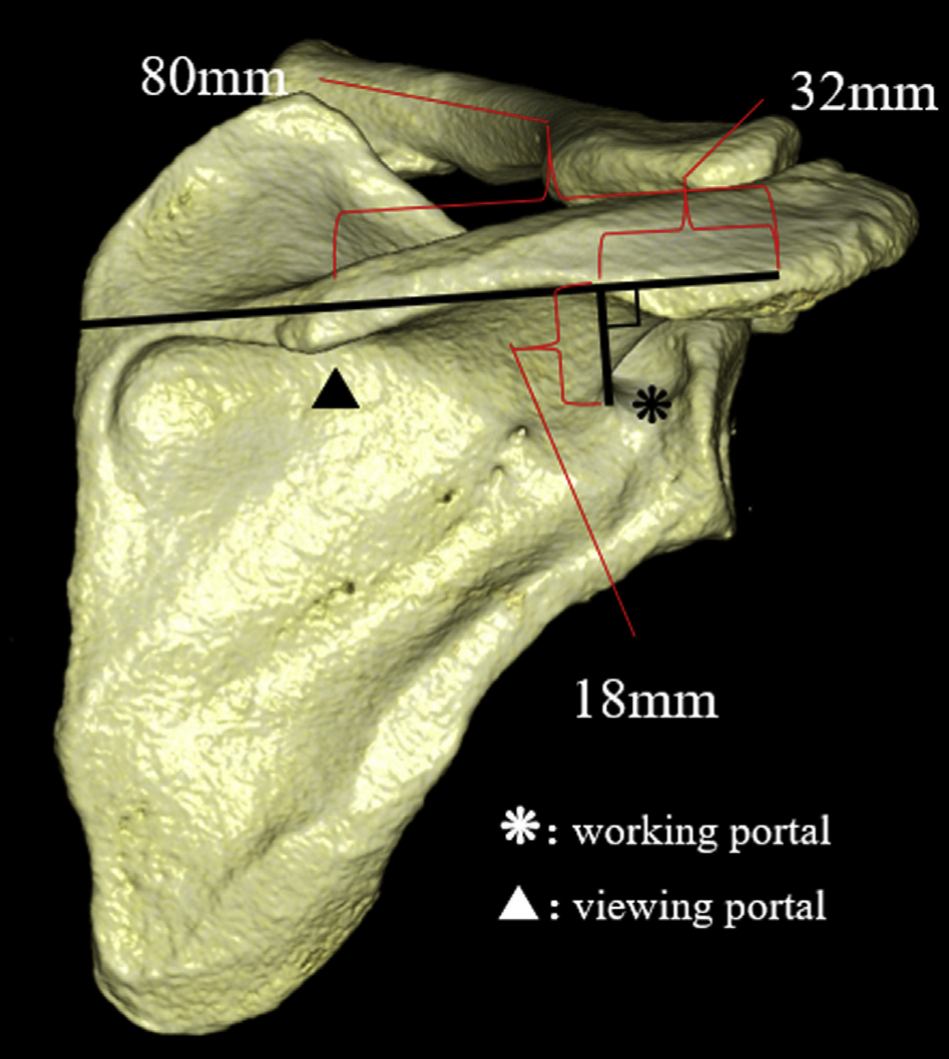
Following the approach used by Plancher et al,^14^ it would be optimal to create a working portal positioned 32 mm medial to the posterolateral corner of the acromion and approximately 18 mm inferior to the scapular spine and a viewing portal 80 mm medial to the posterolateral corner of the acromion.

Plancher et al^14^^,^^15^ reported that it would be useful to create a working portal, which allows for manipulation of the spinoglenoid ligament, 40 mm medial to the posterolateral corner of the acromion. The distance from the posterolateral corner of the acromion to the spinoglenoid notch was approximately 32 mm in this study, which generally supports their results. In addition, our study demonstrated that the distance from the scapular spine to the spinoglenoid notch was 18 mm. Following the approach used by Plancher et al,^14^ it would be optimal to create a working portal positioned 32 mm medial to the posterolateral corner of the acromion and approximately 18 mm inferior to the scapular spine and a viewing portal 80 mm medial to the posterolateral corner of the acromion (Fig. 6).

### Limitations

This study has several limitations. First, there may be numerous factors that affect the morphology of the scapula. This study included only the Far East Asian population. The values obtained for d 1–8 may differ among races, and because our study evaluated relatively older individuals, the values may also differ among other age groups. Second, the height of elderly individuals may be lesser due to the deterioration of spinal alignment. Third, this study lacked interobserver and intraobserver reliability of the measurement. In addition, because this study was based on CT bony measurements, there may be errors from the actual body surface measurements, especially in obese patients. Moreover, we did not confirm these measurements arthroscopically or using a cadaver; thus, their validity was limited. Nevertheless, this study demonstrated that the distances from the posterolateral corner of the acromion to the suprascapular and spinoglenoid notches were not correlated with height and may be considered indicators for creating portals.

## Conclusions

Regardless of height and sex, the distances from the posterolateral corner of the acromion to the suprascapular (medial-lateral direction) and spinoglenoid (medial-lateral direction) notches were approximately 43 and 32 mm, respectively. Therefore, creating portals at these locations may be effective for arthroscopic suprascapular nerve decompression.

## Disclaimers:

Funding: No funding was disclosed by the authors.

Conflicts of interest: The authors, their immediate family, and any research foundation with which they are affiliated did not receive any financial payments or other benefits from any commercial entity related to the subject of this article.
